# Meet the Medicines—A Crowdsourced Approach to Collecting and Communicating Information about Essential Medicines Online

**DOI:** 10.3390/ijerph20054242

**Published:** 2023-02-27

**Authors:** Yaela N. Golumbic, Kymberley R. Scroggie, Ciara R. Kenneally, Jiarun Lin, Mitchell T. Blyth, Genevieve Firmer, Peter J. Rutledge, Alice Motion

**Affiliations:** 1 SCOPE Research Group, School of Chemistry, The University of Sydney, Sydney, NSW 2006, Australia; 2 Charles Perkins Citizen Science Node, The University of Sydney, Sydney, NSW 2006, Australia; 3 The Steinhardt Museum of Natural History, Tel Aviv University, Tel Aviv 6997801, Israel; 4 Drug Discovery Institute, The University of Sydney, Sydney, NSW 2006, Australia; 5 Research School of Chemistry, Australian National University, Canberra, ACT 2601, Australia

**Keywords:** citizen science, science communication, essential medicines, social media, accessibility

## Abstract

The World Health Organization (WHO) maintains a list of medicines and medical devices, *essential medicines*, that should be available to everyone, to form a functioning healthcare system. Yet, many of these medicines remain out of reach for people around the world. One significant barrier to improving the accessibility of *essential medicines* is a paucity of information about both the extent and causes of this problem. E$$ENTIAL MEDICINE$ (E$$) is a citizen science project designed to investigate this deficit of information by recruiting members of the public to find, validate, compile and share information on *essential medicines* through an open, online database. Herein, we report an approach to crowdsourcing both the collection of information on the accessibility of *essential medicines* and the subsequent communication of these findings to diverse audiences. The Meet the Medicines initiative encourages members of the public to share information from the E$$ database, in a short video format appropriate for social media. This communication details the design and implementation of our crowdsourced approach and strategies for recruiting and supporting participants. We discuss data on participant engagement, consider the benefits and challenges of this approach and suggest ways to promote crowdsourcing practices for social and scientific good.

## 1. Introduction

Advancements in modern medicine have led to people live longer and healthier lives than ever before, yet over 2 billion people do not have access to the medicines they need [1]. The starkness of this inequality has been recognized by both the United Nations (UN) and the World Health Organization (WHO), with the former citing increased coverage and accessibility to medication as a key priority in achieving Sustainable Development Goal (SDG) 3 (https://www.who.int/europe/about-us/our-work/sustainable-development-goals/targets-of-sustainable-development-goal-3, accessed on 20 January 2023): a world with ‘good health and wellbeing’. In order to reach this goal by 2030, a number of targets have been outlined including one that aims to achieve universal health coverage, access to safe, effective, high quality and affordable essential medicines and vaccines for all (3.8) [2]. The WHO has also placed significant emphasis on the importance of medicine accessibility, most notably through the biannual publication of the Model Lists of Essential Medicines since 1977, which highlights medicines that “satisfy the priority health care needs of the population” [3].

The 22nd Model List of Essential Medicines [4], published in 2021, includes 479 medicines and medical devices indicated for the prevention, management and treatment of health conditions such as cancers, cardiovascular diseases, pain and endemic diseases. Within a functioning healthcare system, the WHO states that *essential medicines* should be available at all times, in sufficient amounts, in appropriate dosage and form, of assured quality, with adequate information and at affordable prices. The list is intended to guide governments in making decisions regarding which medicines should have regulatory approval on a national level (authority to be prescribed and sold), as well as economic support such as government subsidies, and to lead to improved accessibility [5].

Unfortunately, the WHO’s vision is not yet a reality for much of the global population [5,6]. The reasons for this are multifaceted and may include long-term challenges such as geographical location (e.g., physical distance from urban areas), low market incentives (exacerbated by few patients living with a condition, high manufacturing costs and/or low profit margins) and other societal challenges such as cultural, political or religious influences [7].

On a short-term scale, world events can also severely affect the availability of medicines. A recent example were the premature claims that hydroxychloroquine (an *essential medicine* first patented in 1955 for the treatment of malaria and now widely used for the treatment of systemic autoimmune diseases [8]) was an effective treatment for COVID-19. This contributed to shortages in supply [9,10] and a resultant increase in cost. While evidence has now been published that discredits such claims [11] and the FDA has revoked its emergency-use authorization [12], the surge in demand and price impacted patients who rely on hydroxychloroquine to treat autoimmune conditions [13].

Arguably the biggest contributor to the inaccessibility of *essential medicines* is their price, with some experiencing large hikes. Infamously in 2015, Daraprim (active ingredient pyrimethamine), an off-patent medication discovered in 1952, increased by over 5000% from 13.50 USD per tablet to 750 USD [14]. It is not only *essential medicines* that are affected, however. A recent report by the Assistant Secretary for Planning and Evaluation [15] found that over the 2021/2022 financial year, the price of 1216 products increased above that of inflation (8.5%) with an average price increase of 31.6% in the USA. One of the most significant barriers to improving the accessibility of *essential medicines* is the paucity of information about the extent of the problem. The absence of aggregated, transparent and publicly accessible information means system-wide understanding and decision-making is impaired, and opportunities for policy decisions that can positively impact medicine accessibility are limited. For example, in the case of Daraprim, the monopoly over manufacturing rights in the USA was not common public knowledge (though the information was publicly available, for those specifically searching for it). This highlights the importance of creating transparent and accessible databases to aggregate information, which is otherwise scattered, inconsistent, unreliable and often difficult to find.

Following the Daraprim price hike, a number of steps have been taken to remedy the significant inequalities in the accessibility of medicines. Alpern et al. [16] identified several medicines on the WHO’s Model List of Essential Medicines that had only 1–2 approved manufactures in the USA, 9 of which had also seen a price increase and 17 that they deemed were at risk of a price increase. The FDA now maintains a list of off-patent, off-exclusivity drugs (See—https://www.fda.gov/drugs/abbreviated-new-drug-application-anda/list-patent-exclusivity-drugs-without-approved-generic accessed on 20 January 2023) without an approved generic alternative, and to control price increases, announced the Drug Competition Action Plan in 2017 [17].

E$$ENTIAL MEDICINE$ (E$$; See—https://www.breakinggoodproject.com/essentialmedicines accessed on 20 January 2023) is a citizen science project with the objective to better understand accessibility to *essential medicines* and identify attributes that contribute to their inaccessibility [18]. This is achieved by engaging volunteers in online searches to locate relevant information about *essential medicines*, scaffolded across three challenges designed by the E$$ team. The data collected by citizens contributes to the creation of an open database which aims to bring to light trends that lead to the inaccessibility of a medicine and identify medicines at risk of becoming inaccessible before it occurs. The database is an open tool we hope will support informed decision making by researchers, policy makers, patients, and other interested parties.

E$$ is part of the broader Breaking Good initiative, which aims to empower members of the public to become active contributors to projects that improve human health. Breaking Good has worked with young people to recreate expensive medicines such as Daraprim and participate in drug discovery projects for mycetoma and malaria [14], and E$$ has now expanded this work beyond a laboratory setting. The E$$ project was developed in part due to the COVID-19 pandemic, at a time when conversations about medicines and their availability were thrust into the spotlight. The world looked to scientists to develop vaccines to prevent infection and explore possibilities of novel curative medicines, and medicines that reduced the severity of symptoms for patients. This challenging time created the ideal setting for a citizen science project aimed at encouraging greater public scrutiny of, and discussion around, the accessibility of medicines. In particular, the setting created by the COVID-19 pandemic demonstrated the importance of the timely, accurate and comprehensible communication of science to the public.

As a field of research and practice, science communication aims to enhance the public’s scientific awareness, understanding, literacy and culture [19]. At its most effective, science communication informs people about the causes, effects, benefits and risks of science-related topics, enabling them to make informed decisions in their everyday lives [20]. Unfortunately, learning to communicate with the public has not historically been part of a scientist’s training and is not a skill that comes naturally to all scientists, nor one that is a prerequisite for researchers [21,22]. Concerns regarding the time required to conduct science communication, and beliefs about communication ‘taking over’ the more traditional aspects of a researchers’ role, are also prevalent among scientists [23,24,25]. This can make rapid and effective science communication challenging, and impact public awareness of science; for example, challenges in communicating emerging information and risk–benefit profiles of novel vaccines as evidenced during the COVID-19 pandemic [26] (The authors also note the extraordinary skill and successful efforts of many scientists and researchers who communicated their research and that of others throughout the COVID-19 pandemic).

Recent models of science communication indicate the effectiveness of a two-way dialogue between scientists and the public, where both groups can benefit from listening to and learning from each other [27,28]. Of particular note is citizen science, which encourages wider participation in scientific inquiry by engaging the public in tasks from data collection to data analysis [29]. Citizen science is therefore emerging as a useful method to support a new relationship between scientists and the public, while encouraging the active participation of the public in science and creating a space where scientific and non-scientific knowledge can co-exist and reinforce one another [30,31]. Projects such as E$$ demonstrate the power of combining social understanding and scientific knowledge in an attempt to answer challenging problems. Yet, even within citizen science, communicating with the public is not without challenges. Research highlights the tension between scientists’ scientific and public engagement goals and the delicate balance needed to deliver benefits in both domains, while also maintaining ongoing communication and sustaining the project reach [32].

### Aims of This Paper

To address gaps in (1) the understanding of medicine accessibility and (2) challenges with science communication around this topic, we developed a crowdsourcing strategy for both the collection of information about medicine accessibility (E$$), and the sharing of these findings with the public (Meet the Medicines). This communication describes the design and implementation of our crowdsourcing approaches, including recruitment strategies and the support provided to participants during involvement. This paper also shares insights into participant engagement in both aspects of the project, and the reach of communication outputs. Finally, we discuss the benefits and challenges of our approach to promote crowdsourcing practices for social good, health and well-being.

## 2. Methods

### 2.1. Crowdsourcing Data Collection: E$$ENTIAL MEDICINE$

**Figure 1:**
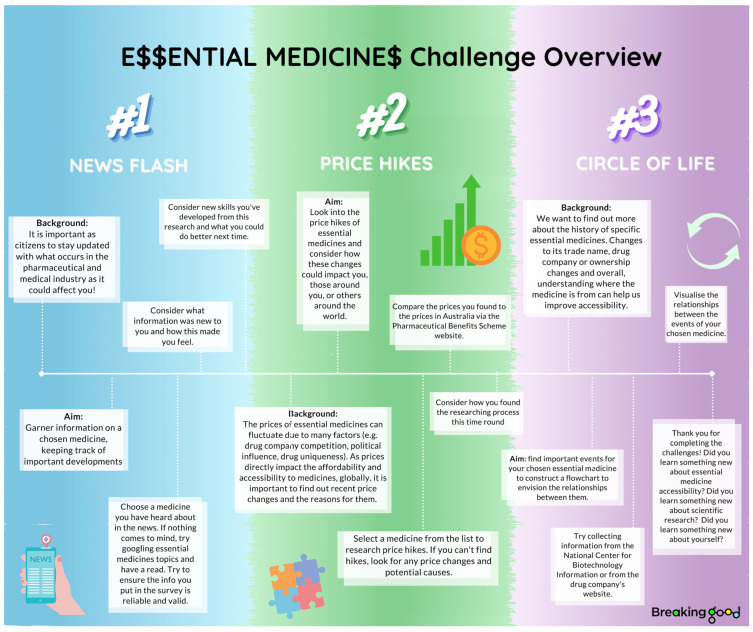
E$$ challenges overview provided for participants.

**Figure 2:**
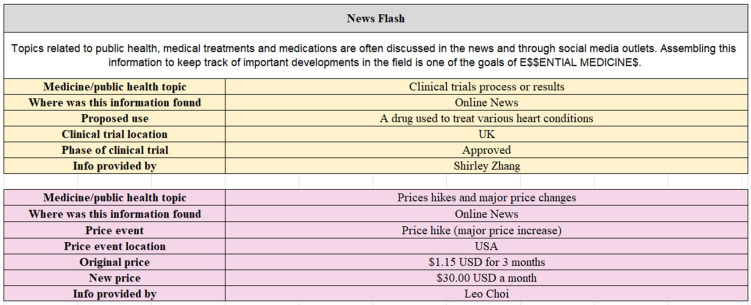
Printscreen of the E$$ ‘News Flash’ challenge database presenting public health and medical information featured in the news for Digoxin.

E$$ employs crowdsourcing to collaboratively search, review and gather web-based data on the accessibility of *essential medicines*. Stage I of the project explores the approval of these medicines in countries around the world, first through manual searching, and now through participant-generated automation. Stage II introduces three challenges: News Flash, Price Hike$ and Circle of Life, guiding participants through a scaffolded and systematic web exploration to investigate aspects of medicine accessibility (Figure 1) [18]. The information collected by participants in these challenges is collated and sorted in a database that abides by FAIR (Findability, Accessibility, Interpretability and Reuse of digital assets) [33] and open science principles (Figure 2) [34].

To recruit participants, the E$$ team utilizes a range of strategies. The project’s initiation was scheduled during Australia’s National Science Week in 2020 and involved a panel discussion on the accessibility of *essential medicines* followed by an E$$ hack-a-thon. Further participation was encouraged online through Breaking Good’s social media, website and GitHub, and quarterly newsletters. In 2020 and 2021, we created and hosted school workshops for year 9 and 10 students. Following the success of these workshops, the program is now transitioning into a curriculum-embedded unit for year 9 and 10 science students.

### 2.2. Crowdsourcing Communication: Meet the Medicines

Meet the Medicines is a crowdsourced social media campaign which communicates data collected in E$$ to the public through short videos. The videos were created by both existing E$$ participants and new recruits, who used a step-by-step guide to transform E$$ data into an engaging communication format, which was then posted on social media.

**Figure 3:**
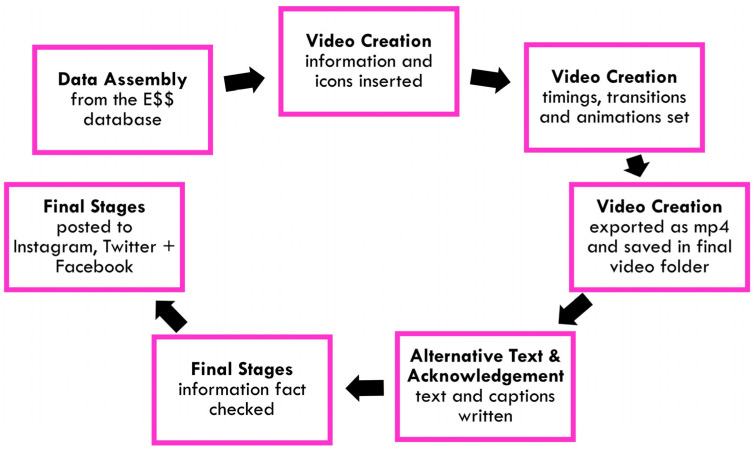
Workflow of the crowdsourced social media campaign from data assembly to posting on social media platforms.

The criteria for our chosen video creation program were that it should be: simple to use, available to a wide audience and freely available. The well-known and widely used Microsoft PowerPoint program (Redmond, WA, USA) paired with the free Google Slides app (Mountain View, CA, USA), and the ability to easily convert between formats for editing by participants, satisfied these criteria. A resource pack was created (JL) to facilitate video creation, which included a PowerPoint template with a standard storyboard and preprogramed animations, and an of over 600 icons illustrating different types of medication and disease. A detailed user guide for participants provided step-by-step instructions on how to use the resources. This allowed for the simple, modular creation of videos for different *essential medicines*. The creation of these videos followed three steps (see Figure 3):

1. Participants complete a Google Sheet for an *essential medicine* of their choice, detailing the information they would like to include in their video (e.g., conditions the medicine is used to prevent, manage and/or treat, the general route of administration, accessibility issues, etc.) taken from the E$$ database.
2. Participants complete a PowerPoint template choosing the colour scheme and icons from the Icon Pack, write captions and alternative text, adjust timings and export the PowerPoint in video format.
3. Following a fact check by the project’s principal scientist (KRS), the final video is disseminated via social media by the project’s management team. Each participant who aided in the creation of the video is credited through their social media handles, with the option to remain anonymous.

All resources participants use in the creation of these videos are stored within a folder on Breaking Good’s Google Drive (https://drive.google.com/drive/folders/13crP2Guh1HIdiaa90tjr6F4b0QJuyJ-W?usp=sharing, accessed on 20 January 2023) and have been shared through GitHub (https://github.com/TheBreakingGoodProject/Essential-Medicines/wiki/Meet-the-Medicines!, accessed on 20 January 2023) and the website. The final videos are saved in this folder for easy access by other participants and the project management team. Google Drive was chosen to lower the barrier to participation, as all documents and icons can be duplicated and edited online, meaning that only an email address and internet connection are required to create a video (removing the need for additional software).

Volunteers were recruited via: workshops with researchers and professional staff at The University of Sydney, integrated undergraduate research programs and social media invitations.

## 3. Results

### 3.1. Creating an Essential Medicine Accessibility Database

#### 3.1.1. Database Stage I: Regulatory Approvals

The first unambiguous barrier to medicine accessibility is approval for it to be provided and sold. Each country has its own regulatory body which has the authority to approve or decline the sale of new medicines products. To begin understanding the factors that influence *essential medicine* accessibility, it was important to determine if they had regulatory approval or not. Stage I of E$$ investigated *essential medicine* accessibility in the USA and their regulatory approval by the U.S. Food and Drug Administration, with participants manually entering information from the Food and Drug Administration’s Approved Drug Products with Therapeutic Equivalence Evaluation publication (colloquially, the Orange Book) into an online form. Participants were provided with a screen share instructional video to explain how to navigate the Orange Book database, what data was in the database and which to collect. This was embedded within the online form along with written explanations for each required entry. The data collected in this stage is shown in Table 1.

During the process of crowdsourcing these data, participants suggested using automated extraction to speed up data collection, and a script was co-designed (with significant contribution from MTB); see discussion on GitHub (https://github.com/TheBreakingGoodProject/Essential-Medicines/issues/2, accessed on 20 January 2023) for more detail. The script collected the same data as was collected manually (see Table 1) and addressed repetition challenges observed for those medicines with approved generics by combining these data into a single entry in the outputted Excel spreadsheet. Running the script written by participants and verifying the output with data manually collected by 62 participants (129 entries), the project was able to quickly generate a database (https://doi.org/10.6084/m9.figshare.12525965.v1, accessed on 20 January 2023) of all the anti-infective *essential medicines* approved in the USA. The project is currently expanding the database to include all *essential medicines* with an open invitation for participants to optimize the script, clean the retrieved data and incorporate this into the current database.

While the automated process accelerates data collection from a particular public resource, it is not currently scalable; suitable resources on regulatory approvals need to be identified for each country and require the development of bespoke scripts to automate data extraction. To date, participants have contributed a second script that automates extraction of information from the Therapeutic Goods Administration’s eBusiness Service (https://www.ebs.tga.gov.au/, accessed on 20 January 2023) database and Australian Register of Therapeutic Goods (https://www.tga.gov.au/resources/artg, accessed on 20 January 2023), which has been included in a E$$ database on approved *essential medicines* in Australia (https://doi.org/10.6084/m9.figshare.21637529.v1, accessed on 20 January 2023). The project is now looking to expand this process to additional countries.

#### 3.1.2. Database Stage 2: The Challenges

Stage II of the project involves an interrogation of the more nuanced barriers to accessibility. Participants contribute this information to the project’s database through the News Flash, Price Hike$, and Circle of Life challenges. Each challenge was designed to stand alone, with differing aims and time commitments to support different interests and levels of participation. An overview of the challenges is provided at the beginning to help participants understand the aims and what is involved in each challenge (see Figure 1) and explanations of each challenge are embedded within the data collection platform, guiding participants step-by-step.

News Flash is a rapid-fire challenge that keeps track of current affairs and media influences with participants submitting updates on medicines as reported in news articles or in the media more broadly. Price Hike$ investigates historical and current medicine pricing to track changes over time. It involves participants using search engines to find pricing information on a medicine of their choice. Tips on how to choose and evaluate key resources, and refine searches, are provided within the challenge to aid meaningful contribution. The final challenge, Circle of Life, is an open-ended challenge which explores the history of the *essential medicines*. Participation involves use of search engines to find important events in the medicine’s life cycle such as discovery, acquisitions, shortages and new indications, with flexibility to explore aspects that interest them. Again, tips on conducting online searches were provided along with a complete example for the medicine cycloserine and a template to aid the visualization and collation of the information found.

The data collected through these challenges is unstructured, and diverse in terms of detail and certainty, so careful database design was required to ensure its readability and usability. The database was co-designed by undergraduate students at The University of Sydney, following participation in the challenges and a scaffolded process of self-reflection [14]. Key elements of the updated database design included improving its readability, reducing duplicity, documenting contributors and integrating the principles of FAIR data and open science. A pilot database featuring information on 17 of the *essential medicines* has been shared to FigShare (https://doi.org/10.6084/m9.figshare.21637559.v2, accessed on 20 January 2023)—an example of the database structure can be seen in Figure 2. The project aims to expand this database to all *essential medicines* in the future, working iteratively to update the database as information accumulates.

#### 3.1.3. Database Stage 3: The Future

As participation has increased and the database expanded, maintaining consistency in the consolidation of the structured and unstructured data into a single FAIR database, has proved challenging. While the E$$ database design has been upgraded to a more readable and accessible format, databases from different stages of the project remain separate. At a minimum, the future aim of the project is the automation of real-time data input into a single public-facing, easily searchable database. E$$’s ultimate objective is to create a live dashboard which shares information collected from all the project stages. A dashboard is envisioned for each of the *essential medicines*, including visualizations such as a timeline and an interactive world map detailing information on medicine approval and price tracking. These would support consistency in the structure of data, ensure timely dissemination of project outputs and streamline access to and the use of the information collected in E$$ by researchers, policy makers, patients, etc., to foster informed decision making, lobby for greater accessibility of medicines and support further research.

### 3.2. Meet the Medicines Communication

Concurrently, we implemented a crowdsourced communication campaign for E$$ data. Over the course of seven months, 23 Meet the Medicines videos were created by 27 volunteers. The volunteers contributed to the assembly of information from the E$$ database into a dedicated spreadsheet and the creation of Meet the Medicines videos. Each video shared accessibility data and fun (or useful) facts about one *essential medicine*, and were released regularly on social media (Instagram and Twitter, both @_breakinggood and Facebook, @thebreakinggoodproject).

At the time of paper submission, 17 videos had been published. Videos have been published for the following medicines: acetylsalicylic acid (Aspirin), povidone iodine (Betadine), pyrimethamine (Daraprim), salbutamol (Ventolin), doxycycline (Vibramycin), pyrazinamide (Tebrazid), loperamide (Imodium), rifampicin (Rifadin), ketamine (Ketalar), rivaroxaban (Xarelto), fluoxetine (Prozac), warfarin (Coumadin), emtricitabine + tenofovir (Pre-Exposure Prophylaxis, PrEP), blood, naloxone (Narcan), levonorgestrel + ethinylestradiol (also known as the pill) and levodopa + carbidopa (Sinemet).

These 17 videos have received a total of 7628 views, averaging 449 views per video, across the three social media platforms (see Table 2). Posts received an average of 39 likes, 10 shares and five comments. Twitter was the most popular platform for views and sharing in the form of retweets; however, Instagram received more engagement with likes and comments. Interestingly, two videos received the most views on Facebook; levonorgestrel + ethinylestradiol (also known as the pill) and rifampicin (an antibiotic used for the treatment of tuberculosis, (TB)) with 940 and 912 views, respectively. This may be due to the prevalence and familiarity with the pill (levonorgestrel + ethinylestradiol) as a popular means for contraception. In the case of the rifampicin video, the fact that the creator (@Klemistry) is an active researcher in open-source drug discovery for tuberculosis (TB) (@OpenSourceTB), who shared the video to their TB-focused social networks, has likely contributed to broader engagement.

### 3.3. Meet the Medicines: Beginning to End

To demonstrate the process of creating a Meet the Medicines video from data collection to dissemination, we provide a detailed example using the *essential medicine* rifampicin (an antibiotic used for the treatment of TB). We describe the process undertaken by a volunteer, from data aggregation through to video creation, and include information on the publication and engagement with the video (summarized in Figure 3).

#### 3.3.1. Data Assembly

The volunteer who chose to investigate rifampicin (@Klemistry, an honours student in the School of Chemistry at The University of Sydney, researching potential new TB medicines), started by assembling data from E$$’s database, identifying the year the medicine was first marketed (1968), the disease the drug was marketed to treat (TB), the dosage forms (oral) and common names (Rifampin). Next, they searched for interesting and engaging facts about the medicine, and recorded all the information collected, along with sources, in a shared Google Sheet (see Table 3).

#### 3.3.2. Video Creation

**Figure 4:**
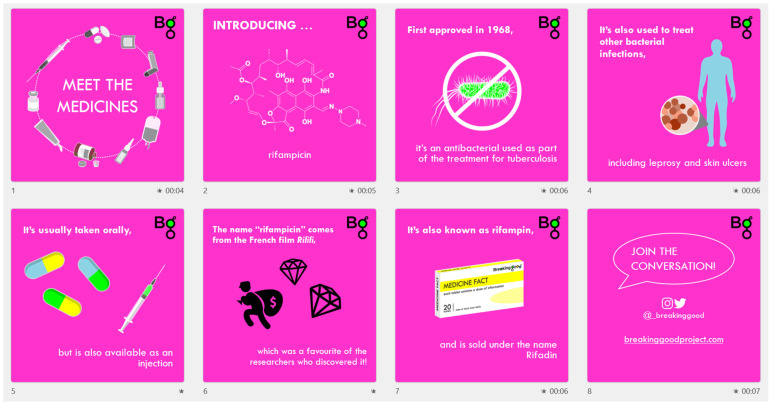
Example slideshow created for rifampicin.

Following data assembly, the volunteer downloaded the PowerPoint template, inserted the information collected and matched this with relevant icons from the Meet the Medicines Icon Pack (and where needed, other free icon websites) to create a slideshow (see Figure 4). Once slides were completed, timings, transitions and animations were set for the creation of a video which was then exported as an mp4 file.

#### 3.3.3. Alternative Text and Acknowledgments

**Figure 5:**
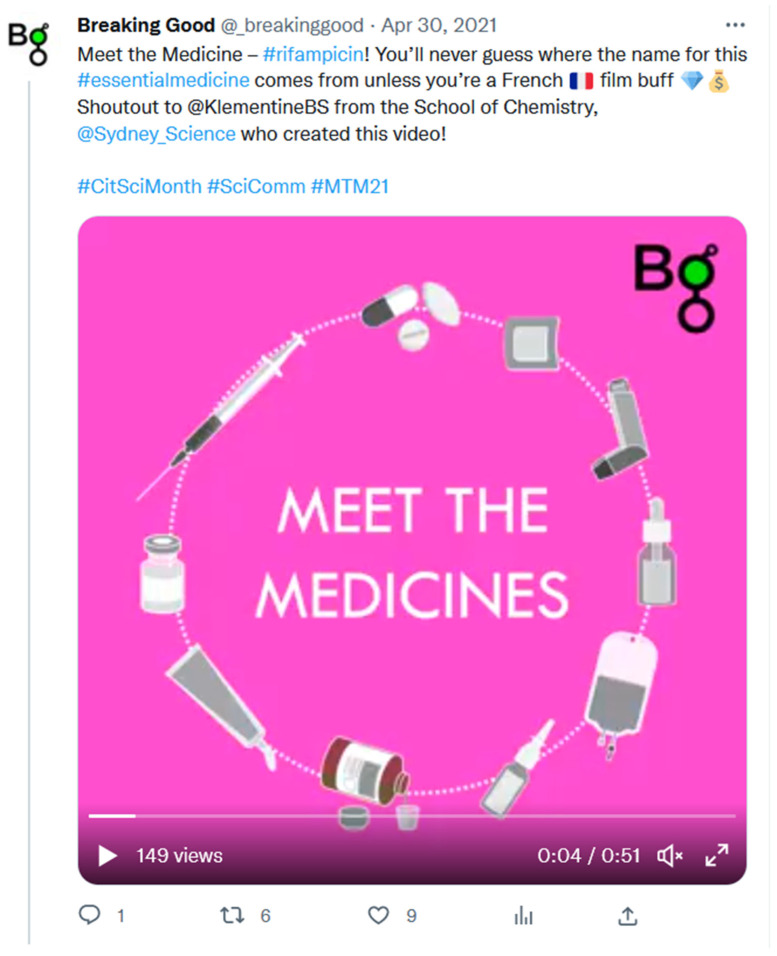
Published tweet for rifampicin video. See—https://twitter.com/_breakinggood/status/1387997091641794562?s=42&t=YQlFlekFEt9A3UK_zaUFRg, accessed on 20 January 2023.

To increase the accessibility of the videos, the volunteer wrote alternative text (alt text) which was embedded in each slide. They also wrote an engaging caption for their video, designed to be used as part of the social media post, including an acknowledgement and their social media handles, as seen in Figure 5.

#### 3.3.4. Fact Checking and Posting

Once all stages were complete and marked as so in the tracking documents, the video was fact checked by the Breaking Good team. The video was then posted to Facebook, Instagram and Twitter, with the caption and alt text written by the volunteer (see Figure 5). The Breaking Good team monitored social media for interaction with the content.

## 4. Discussion

This paper describes the crowdsourcing approach employed in E$$ for the collection of information on the accessibility of *essential medicines* and the communication of these data through a social media campaign, Meet the Medicines. Crowdsourcing allowed the team to gather a larger, more diverse set of data than otherwise possible through insourcing methods, while simultaneously expanding the roles of citizen scientists in the project. The idea of fostering diversity in crowdsourcing and citizen science has been discussed at length both as a method for promoting data diversity and as an opportunity to advance public inclusion in science [35,36]. While this commentary presents one project and its multiple uses of crowdsourcing, this idea can be translated to other projects to advance and diversify scientific research.

The larger, more diverse data set collected in E$$ through crowdsourcing, was supported by the willingness to incorporate suggestions from participants with different skill sets. For example, following the suggestion and significant contribution to automating structured data collection efforts made by MTB, an E$$ volunteer, the project significantly progressed, transforming the crowdsourcing capacity to the collection of diverse unstructured data, implemented in Stage II. Similar contributions have been reported in Eterna, an online game for determining RNA molecules structures [37]. Game players collaborated with game developers to improve the game software and discuss their thoughts and ideas for updating the game. This demonstrates the power of crowdsourcing beyond the collective contribution of the masses and the changing of roles from project participant to facilitator.

Crowdsourcing unstructured information is inherently diverse, with individuals’ personal experiences and choices guiding data collection. Here, through community effort, information on the accessibility of *essential medicines* from various online websites and resources was pooled together to form a distinct and diverse database. The choices participants made regarding the keywords used in search engines, their search history and algorithms linked to their social media accounts all lead to a greater diversity in the data collected. This diversity is necessary for the project to achieve its goal of understanding global accessibility to *essential medicines*.

An exciting opportunity to foster data diversity through crowdsourcing is the use of artificial intelligence (AI). AI can aid in the collection of unstructured data, such as those collected in the challenges, facilitate a more systematic searching approach and mediate diverse perspectives. However, assessing information relevance, reliability and implication is an ambitious task for AI, which currently demands human capabilities. Attempts at using unsupervised and semi-supervised machine-learning methods to categorize Tweets on mental health, showed firsthand that there are challenges in quantifying and categorizing unstructured data [38]. AI based on supervised machine learning is more successful in this area and has been used to identify new patterns, for example, in the evolution of neurological disorders [39]. Ultimately, the role and opportunity of AI in research relies on humans working with and understanding it. Such human capabilities will, with no doubt, increase over time and expedite the use of AI for such purposes [40].

Communicating the information collected in citizen science projects is a crucial, yet challenging part of leading such a project. Crowdsourcing the communication through Meet the Medicines videos served as a solution for many of these challenges. First, it streamlined the dissemination of the data collected within the project, ensuring that it was shared with the wider community in an accessible, relevant and engaging way. The videos served to raise public awareness of medicines and to encourage conversations about their discovery, manufacturer, distribution and accessibility. While the example provided above (rifampicin) highlights a fun fact about the medicine, other videos may discuss issues of accessibility, reach and pricing. For example, the videos created for Daraprim (https://twitter.com/_breakinggood/status/1381782394043199489, accessed on 20 January 2023), which discussed the price hike described above, and emtricitabine + tenofovir (PrEP) (https://twitter.com/_breakinggood/status/1400655385288007680, accessed on 20 January 2023), which highlights the increase in its accessibility following its patent expiration in 2020 and the induction of generics. Second, it diversified the roles citizen scientists undertook in the project and provided participants with a choice of contributing to several different activities. A particularly significant contribution to the project was provided by JL, an E$$ volunteer, who designed the Meet the Medicine resource pack and icons, and assisted with the delivery of workshops on video creation. Adding choice to citizen scientist tasks has been suggested as a way to increase participant diversity, by empowering individuals to choose tasks that best fit their skills and interests [31,41]. This in turn serves an additional goal of reducing barriers for participation in citizen science, which also contributes to more diverse participation [37]. Indeed, using data from E$$ ensured that little to no scientific education was required to contribute to the project, encouraging and enabling participation from a wider range of people. Finally, the crowdsourcing communication activities associated with E$$ provided a model to reduce the ongoing load on project leaders to continuously create engaging scientific content for distribution on social media. This could enable a more balanced allocation of responsibilities in managing citizen science projects and a balancing of ongoing coordination tasks. In turn, this approach may improve the sustainability of citizen science projects, as it reduces a well-reported tension between public engagement, scientific goals and expanding the reach of a project [32,42].

Crowdsourcing the creation of the Meet the Medicines videos was not without challenges, however, and did not resolve all communication issues. While video creation reduced the ongoing work of creating content for distribution on social media, it required significant resources to set up, including the creation of video templates and icons. The campaign also needed oversight for volunteer recruitment, fact checking, video refinement and social media posting. It is not yet clear if the collective time invested in these tasks and resources by project leaders will reduce the overall time investment in communication tasks.

An additional challenge encountered was a reduction in momentum over time. Following the first months of initiation, project leaders were less invested in recruiting volunteers due to competing priorities (finalizing research theses and publications or running school workshops) and some members of the project moved on to new roles, resulting in a lower stream of video creation. Video publication slowed down, finally halting for an extended period. This is evidenced by the fact that only 17 of 23 videos created have been published to social media at the time of this paper’s submission. This experience suggests some level of on-going project management is necessary to support crowdsourced data collection and communication in citizen science projects, as has been similarly suggested by Salmon et al. [43]. Moving forward, the team has identified that targeted data collection events such as those hosted by the Aussie Bird Count (https://aussiebirdcount.org.au/ accessed on 20 January 2023), and Frog ID (https://australian.museum/event/frogid-week-2022 accessed on 20 January 2023), are likely to improve both engagement and the manageability of the workload for the project team. Furthermore, engaging participants on an annual basis through initiatives such as in-curriculum school programs can provide continual participation while also providing educational and social benefits.

The Meet the Medicines campaign utilized three main social media platforms: Instagram, Twitter and Facebook. Twitter and Facebook were found to be more useful for the continual sharing of information, while Instagram offers more engagement from users through likes and comments. This higher engagement on Instagram is consistent with other health and science communication studies which looked at followers’ engagement with posts across different social media platforms [44,45]. While this may be a facet of the different features social media platforms offer (e.g., Instagram does not offer a share/retweet feature that posts to the feed), they nevertheless show the need to consider the objectives of a campaign when choosing which social media platform to use. Twitter or Facebook may be more suited for the perpetual sharing of information, while Instagram may support discussion and consultation. The use of other platforms, such as Snapchat and TikTok, could expand the reach, engage more diverse audiences or target specific demographics. However, adding additional platforms require consideration and adjustment of the communication format. The short, animated videos used in Meet the Medicines, for example, are less suited to TikTok which features live-action videos, or Snapchat which features photographs. This, in turn, would demand greater time investment by the team, a resource which, as discussed, has been recognized as a barrier for the ongoing coordination of the campaign.

## 5. Conclusions

E$$ is a citizen science project building a better understanding of global accessibility to *essential medicines* and identifying factors that contribute to an inequitable access to these medicines. This paper describes the crowdsourcing approach we employed in the project to address gaps in the understanding of medicine accessibility, alongside challenges with science communication around this topic and with diversifying participation in citizen science projects. Our work expanded on a traditional use of crowdsourcing to include not only data collection, but also database design and the dissemination of results and science communication on the accessibility of *essential medicines*. Crowdsourcing these activities offers opportunities for citizen scientists to contribute their time, experiences and skills to new areas within scientific and health research and move projects in new directions. Sharing the load in this way is also a significant step towards improving the sustainability of citizen science and provides a model for future crowdsourcing and citizen science initiatives. Though the initial investment in the case of E$$ was substantial, the benefits in streamlining and extending the reach of science communication efforts, diversifying the range of people who participate in scientific endeavours and achieving greater diversity of data and quality contributions for science are significant. These benefits support a future of using crowdsourcing for social good, health and well-being.

## Figures and Tables

**Table 1:** Data collected from the FDA’s Orange Book database by E$$ participants.

Database Title	Data Inputs
Essential medicine	Medicine from the Model List of Essential Medicines
Active ingredient/s	Multiple active ingredients are separated by ‘;’
Trade name	Name given to a product that is registered by its owner as a trade mark
Applicant holder	The company that has FDA approval to supply the product to the USA
Market status	RX—prescription, OTC—over the counter, DISCN—discontinued
Dosage form	Tablet, capsule, ointment, suspension, injectable, etc.
Administration route	Oral, topical, buccal, injection, etc.
Strength	Expressed as a mass in milligrams, e.g., 200 mg, mass per volume, e.g., 200 mg/mL, or mass equivalents if a base, e.g., 200 mg base
FDA application type	N—new drug applications (innovator), A—abbreviated new drug applications (generics)
FDA application number	Six-digit number
FDA product number	If multiple products are applied for in a single application, each product is assigned a number
FDA approval date	Day/Month/Year
Under patent?	Yes or no
Latest patent expiry date	Day/Month/Year
Exclusivity agreement?	Yes or no
Latest exclusivity expiry date	Day/Month/Year

**Table 2:** Engagement with Meet the Medicines videos on Twitter, Facebook and Instagram *.

Platform	Views	Likes	Comments	Shares/Retweets	Total
Facebook	3041	118	19	17	3195
Instagram	1476	362	48	47	1933
Twitter	3111	177	18	96	3402
Total	7628	657	85	160	8530

* data collected on 14 November 2022.

**Table 3:** Data assembled for the Meet the Medicines video on rifampicin.

Essential Medicine	Year First Marketed	Marketed for	Dosage Form(s)	Common or Trade Names	Fun Fact	References
Rifampicin	1968 (Italy)	antibiotic—TB specifically	oral	Rifampin	Named rifampicin because researchers liked a French movie named “Rififi” about jewel heists and gangs (French slang for brawl)	nature.com/articles/ja2014108

## Data Availability

The databases described in this study are openly available in: Anti-infective essential medicines approved in the USA: https://figshare.com/articles/dataset/AntiInfection_Essential_Medicines_Database_21June20_xlsx/12525965, accessed on 20 January 2023. All approved essential medicines in Australia: https://figshare.com/articles/dataset/Australia_-_All_medicines_database_-_03_Feb_2021/21637529, accessed on 20 January 2023. Accessibility information of 17 of the essential medicines: https://figshare.com/articles/dataset/E_ENTIAL_MEDICINE_Challenge_Database_Pilot_29_Nov_2022/21637559, accessed on 20 January 2023.
